# Antimutagenic Activity of Ethanol Extract of *Rhaphidophora pinnata* (L.f) Schott Leaves on Mice

**DOI:** 10.3390/scipharm85010007

**Published:** 2017-02-15

**Authors:** Masfria, Sumaiyah, Aminah Dalimunthe

**Affiliations:** 1 Department of Pharmaceutical Chemistry, Faculty of Pharmacy, University of Sumatera Utara, Tri Dharma Street, No.5, Medan 20155, Indonesia; 2 Department of Pharmaceutical Technology, Faculty of Pharmacy, University of Sumatera Utara, Tri Dharma Street, No.5, Medan 20155, Indonesia; sumaiyah7777@gmail.com; 3 Department of Pharmacology, Faculty of Pharmacy, University of Sumatera Utara, Tri Dharma Street, No.5, Medan 20155, Indonesia; aminah1@usu.ac.id

**Keywords:** Antimutagenic activity, ethanol extract, *Rhaphidophora pinnata* leaves

## Abstract

*Rhaphidophora pinnata* is suggested to prevent or treat cancer of genetic mutations. In this study, antimutagenic activity of an ethanol extract of *Rhaphidophora pinnata* leaves was evaluated by using a bone marrow micronucleus assay on mice. Male mice (20–30 g) were treated for sevendays with an ethanol extract of *Rhaphidophora pinnata* leaves at a dose of 500, 750 and 1000 mg/kg/day/orally, prior to exposure to cyclophosphamide (i.p. 30 mg/kg), 24 h after the end of the treatment. Antimutagenic activity was determined by the decrease of micronuclei (MN). The results showed that a single administration of all variant doses of the extract had significantly decreased the micronucleus formation in bone marrow cell of mice as compared to the cyclophosphamide group. The ethanol extract of *Rhaphidophora pinnata* leaves had antimutagenic activity against cyclophosphamide-induced gene mutation.

## 1. Introduction

Cancer is a vicious tumor that arises and progresses through the accumulation of successive mutations. It involves the activation of proto-oncogenes and inactivation of tumor suppressor genes which causes out of control proliferation of the cells [1]. Gene mutation can be caused by DNA damage. The presence of micronuclei (MN) in cells is considered as a biomarker of DNA damage. MNs are cytoplasmic chromatin-containing bodies that emerge in the cell asa small satellite nucleus around the cell nucleus due to genomic alteration. The MNs assay is an in vivo or in vitro screening cytogenetic test for assessing the genotoxicity of chemicals in an organism [2,3].

Some plant extracts have anticancer activity due to genetic mutations, such as *Carica papaya*, S*olanum lycopersicum* and *Kaempferia rotunda*, but there is a need to explore them for use as antimutagenic and anticarcinogenic food or drug additives [2,3,4]. *Rhaphidophora pinnata*, a family of *Araceae*, has been used as a traditional medicine for the treatment of bacterial infection, cancer, rheumatism, and cough. Chloroform fraction and an ethanol extract of *Rhaphidophora pinnata* showed cytotoxic activity, proliferation inhibition and apoptosis induction to the MCF-7 cell line in vitro [5]. This is the first study to examine the crude of an ethanol extract of *Rhaphidophora pinnata* leaves as anticancer agents in vivo due to genetic mutations. The antimutagenic activity of an ethanol extract of *Rhaphidophora pinnata* leaves was evaluated by using a bone marrow micronucleus assay on mice.

## 2. Materials and Methods

### 2.1. Materials 

*Rhaphidophora pinnata* leaves were obtained from a local garden in Medan, Sumatera Utara, Indonesia. *Rhaphidophora pinnata* was identified in the Research Centre for Biology, Indonesian Institute of Science, Bogor, and the voucher specimen was deposited in a herbarium. 

### 2.2. Preparation of Extracts 

The dried leaves of *Rhaphidophora pinnata* were ground to a coarse powdered form, and wereextracted by the percolation method using ethanol as solvents. The extracts so obtained were concentrated under vacuum using a rotary evaporator (Stuart, Stone, UK) and dried in a desiccator (Merck, Kenilworth, NJ, USA) until use [6].

### 2.3. Phytochemical Screening 

The phytochemical screening of *Rhaphidophora pinnata* leaves powder and ethanol extract was examined based on class compounds, including alkaloids, saponins, flavonoids, tannins, glycosides, anthraquinone glycosides, and triterpenoids/steroid [7,8,9].

### 2.4. Animals

The study was conductedon twenty-five male mice (4–5 weeks old; 25–30 g body weight). They were kept in good laboratory conditions with temperature (25±2°C), light (12 h light: 12 h dark) and given a standard mouse pellet diet and water ad libitum. All animal experiments were used in this study after getting prior permission from the Institutional Animal Ethics Committee, Department of Biology, Faculty of Mathematics and Science, University of Sumatera Utara with the accession number 120/KEPH-FMIPA/2016.

### 2.5. Micronucleus Assay

Male mice (20–30 g) were divided into five groups; each group containing five animals. The first group was treated with 0.5% Sodium Carboxymethyl cellulose (CMC Na) (control). The second group was treated with cyclophosphamide (i.p.30 mg/kg). The third, fourth and fifth groups were treated for 7 days with an ethanol extract of *Rhaphidophora pinnata* leaves at the dose of 500, 750 and 1000 mg/kg/day/orally. Cyclophosphamide (i.p. 30 mg/kg) was given as a single dose to all of the mice 24 h after being treated with the last administration of the extract. Twenty-four hours later, all of the mice were sacrificed by cervical dislocation and bone marrow cells were collected. Bone marrow cells were removed from muscle and aspirated by flushing with BSA (Bovine serum solution) with a syringe. The solution was centrifuged at 1200 rpm for 5 min. The cells in the sediment were separated with the supernatant. Then, a small drop of the viscous suspension was put on a slide and spread by pulling the material behind a polished cover glass held at an angle of 45°. The preparation was dried on the slide and fixed for 2–5 min. The fixation of cells on the slide was done by rinsing with methanol for 10 min. Then, staining was carried out in Giemsa solution for 10 min. Slides were rinsed in distilled water and cleaned with filter paper on the back side of the slide. Erythrocytes cells were scored for micronuclei under the microscope. At least 1000 cells per animal were scored for the incidence of micronuclei [2,3,10].

### 2.6. Statistical Analysis

The micronuclei data were statistically analyzed using one-way analysis of variance (ANOVA), followed by the post-hoc Tukey test, with a significance level of 95%.

## 3. Results 

Phytochemical screening of the dry material can be used as an early data to determine the compounds that are contained in the ethanol extract of *Rhaphidophora pinnata* leaves. The result showed that the dry material and an ethanol extract of *Rhaphidophora pinnata* contained tannins, flavonoids, alkaloids, glycosides, saponin, and triterpenoid/steroid (Table 1). 

The results of the formation of micronuclei in the bone marrow erythrocytes of mice are depicted in Table 2. There was an increase in the polychromatic cells with micronuclei in cyclophosphamide treated animals (Table 2). It showed that once-daily administration of all varying doses of the extract for sevendays had significantly decreased the formation of the micronucleus compared to the cyclophosphamide group.

## 4. Discussion

In this study, the number of micronuclei was increasing in the cyclophosphamide group. However, it was decreased after the administration of 500, 750 and 1000 mg/kg/day extracts for sevendays. All of the variant doses of the extract showed a significant antimutagenic effect. 

The invivo micronucleus test is a very sensitive method to identify the clastogenic effects of chemicals and drugs or to evaluate the change of the chromosome structure using various alkylating agents such as cyclophosphamide. Cyclophosphamide is genotoxic and damages DNA by oxidative stress, transferring the alkyl cluster to the cells [3,4,10]. Some studies used cyclophosphamide as an inducer to produce micronuclei in bone marrow [2,3,4,10]. The micronucleus is a small chromosome resultingfrom a broken chromosome in the cell that is derived from the anaphase stage and mitosis process. The micronucleus that results in mutation may lead to malignant transformation (cancer) by the multistage process [11].

Based on the result, an ethanol extract of *Rhaphidophora pinnata* contained tannins, flavonoids, alkaloids, glycosides, saponin, and triterpenoid/steroid. In this study, we deduce that flavonoid and other phenolic compounds in this extract are responsible for the antimutagenic effect on mice that are exposed to cyclophosphamide. This compound can prevent DNA damage due to itsability to protect the DNA from free radicals. Some studies have shown that phenolic compounds have biological activity, including antioxidant, antitumor, antimutagenic, and antibacterial properties [12,13,14]. Another study showed that phenolic compounds can be used to prevent various diseases, such as cancer and cardiovascular diseases [15].

## 5. Conclusions

An ethanol extract of *Rhaphidophora pinnata* leavesat a dose of 500, 750 and 1000 mg/kg/day had antimutagenic activity against cyclophosphamide-induced gene mutation.

## Figures and Tables

**Table 1:** Phytochemical screening of the dry material and ethanol extract.

No.	Phytochemical Screening	Dry Material	Ethanol Extract
1	Alkaloids	+	+
2	Flavonoid	+	+
3	Glycosides	+	+
4	Anthraquinone Glycosides	−	−
5	Saponin	+	+
6	Triterpenoid/Steroid	+	+
7	Tanin	+	+

Note : (+) = present ; (-) = absent.

**Table 2:** Effect of an ethanol extract of *Rhaphidophora pinnata* leaves on the micronucleus assay in mice.

No.	Group	Number of Micronuclei/1000 cells ± SEM
1	0.5% CMC Na	20.60 ± 2.96*
2	Cyclophosphamide	55.00 ± 2.95
3	EERPL 500 mg/kg bw	25.20 ± 0.66*
4	EERPL 750 mg/kg bw	15.20 ± 0.66*
5	EERPL 1000 mg/kg bw	9.20 ± 0.66*^,#^

Note: All recorded rates were compared with the control group* (*p* < 0.05) and cyclophosphamide group^#^ (*p* < 0.05). CMC Na: Sodium Carboxymethyl cellulose, EERPL: Ethanolic Extract of *Rhaphidophora pinnata* leaves.
